# 

**Published:** 1882-08

**Authors:** 


### TABLE OF CONTENTS FOR AUGUST, 1882.

ORIGINAL COMMUNICATIONS. PAGE

Art. I.—Homœopathy. By A. B. Lyman, M.D. .......... 505

Art. II.—The American Dental Association .......... 510

SELECTIONS.

Carbolic Acid in Urine .......... 526

The Physiological Action of Blood-Letting .......... 527

Editorial .......... 528

